# Cross-Border Cholera Outbreaks in Sub-Saharan Africa, the Mystery behind the Silent Illness: What Needs to Be Done?

**DOI:** 10.1371/journal.pone.0156674

**Published:** 2016-06-03

**Authors:** Godfrey Bwire, Maurice Mwesawina, Yosia Baluku, Setiala S. E. Kanyanda, Christopher Garimoi Orach

**Affiliations:** 1 Ministry of Health Uganda, Control of Diarrheal Diseases Unit, Kampala, Uganda; 2 Ministry of Health Malawi, Lilongwe, Malawi; 3 Ministry of Health Uganda, Bwera Hospital, Kasese, Uganda; 4 Makerere University School of Public Health, Kampala, Uganda; Institute for Health & the Environment, UNITED STATES

## Abstract

**Introduction:**

Cross-border cholera outbreaks are a major public health problem in Sub-Saharan Africa contributing to the high annual reported cholera cases and deaths. These outbreaks affect all categories of people and are challenging to prevent and control. This article describes lessons learnt during the cross-border cholera outbreak control in Eastern and Southern Africa sub-regions using the case of Uganda-DRC and Malawi-Mozambique borders and makes recommendations for future outbreak prevention and control.

**Materials and Methods:**

We reviewed weekly surveillance data, outbreak response reports and documented experiences on the management of the most recent cross-border cholera outbreaks in Eastern and Southern Africa sub-regions, namely in Uganda and Malawi respectively. Uganda-Democratic Republic of Congo and Malawi-Mozambique borders were selected because the countries sharing these borders reported high cholera disease burden to WHO.

**Results:**

A total of 603 cross-border cholera cases with 5 deaths were recorded in Malawi and Uganda in 2015. Uganda recorded 118 cases with 2 deaths and CFR of 1.7%. The under-fives and school going children were the most affected age groups contributing 24.2% and 36.4% of all patients seen along Malawi-Mozambique and Uganda-DRC borders, respectively. These outbreaks lasted for over 3 months and spread to new areas leading to 60 cases with 3 deaths, CRF of 5%, and 102 cases 0 deaths in Malawi and Uganda, respectively. Factors contributing to these outbreaks were: poor sanitation and hygiene, use of contaminated water, floods and rampant cross-border movements. The outbreak control efforts mainly involved unilateral measures implemented by only one of the affected countries.

**Conclusions:**

Cross-border cholera outbreaks contribute to the high annual reported cholera burden in Sub-Saharan Africa yet they remain silent, marginalized and poorly identified by cholera actors (governments and international agencies). The under-fives and the school going children were the most affected age groups. To successfully prevent and control these outbreaks, guidelines and strategies should be reviewed to assign clear roles and responsibilities to cholera actors on collaboration, prevention, detection, monitoring and control of these epidemics.

## Introduction

Cholera is preventable and treatable disease however, it remains a major public health problem in many Sub-Saharan African countries causing deaths and retarding development [1–5]. Cross-border cholera outbreaks are common, causing massive suffering, challenging to prevent and control yet very few studies have documented these experiences. Though, knowledge on stopping cholera transmission and deaths is well documented and was used successfully by countries in South America to eliminate cholera during the previous decades [6] this has not been replicated in Africa.

Interventions for cholera prevention and control include provision of good sanitation, safe water, hygiene, health education, surveillance, treatment of the patients, and recently vaccination with the World Health Organization (WHO) recommended vaccines [7,8]. It should be noted that many cholera affected countries in Sub-Saharan Africa have no structured strategy to prevent and control cross-border cholera outbreaks. Often affected countries respond individually to situations requiring joint country efforts. The consequences are protracted epidemics, unnecessary suffering of the populations, economic lose and social disruption.

On the other hand, for a long time, the Asian sub-continent was the home of cholera [9,10]. However, in recent years most of the reported cholera cases have been from Sub-Saharan Africa which contribute 60% of all reported cases and deaths globally [11,12]. Large cross-border cholera outbreaks are common in Sub-Saharan Africa [13]. The affected countries report these outbreaks to WHO to meet their respective country obligation [14] however, very little is done between the affected countries to collaborate in the prevention and control efforts.

Majority of cholera affected countries in Africa subscribe to WHO and use Integrated Disease Surveillance and Response (IDSR) strategy to prevent and control cholera outbreaks [15]. While IDSR has been useful in improving the reporting and response to cholera epidemics in many countries, this strategy does not adequately guide them to address the rampant cross-border cholera outbreaks. Thus these outbreaks persist in some Sub-Saharan Africa countries spreading to new areas, causing ill-health and deaths [16].

Due to several reasons known and unknown, countries in the Great lakes region of Africa are among those with the highest cholera burden [17]. In 2013, a total of 25,762 cholera cases and 490 deaths were reported from WHO African region with the three countries namely DRC, Angola and Mozambique contributing 79.4% of cases and 89% of reported deaths [18].

To reverse the status quo, the current approach has to be reviewed and lessons of the good actions in South America to prevent cholera outbreaks copied. The introduction of the new Oral Cholera Vaccines (OCV) which works synergistically with the other known cholera prevention interventions and are recommended for use by the WHO in cholera prevention in endemic cholera setting for preemptive vaccination for highly at risk communities [19] is another added opportunity to explore.

However, since the production and the stock of these new vaccines is limited, efficient and effective use of the available vaccine stocks is highly recommended by WHO. Therefore, for the Sub-Saharan cholera endemic countries to qualify to use these vaccines, accurate data and documentation on the affected population is a necessity. In the case of the cross-border endemic setting, collaboration of the two neighboring states is very important if successful cholera prevention and control is to be achieved [20].

The objective of this article is to share experiences and challenges for the prevention and control of cross-border cholera epidemics in Sub-Saharan Africa. The country experiences from the two major Africa grouping zones of Eastern and Southern Africa represented respectively by Uganda and Malawi are shared. Most importantly, the authors give recommendations for prevention and control of future and ongoing cross-border cholera outbreaks in Africa and beyond.

## Materials and Methods

**Fig 1:**
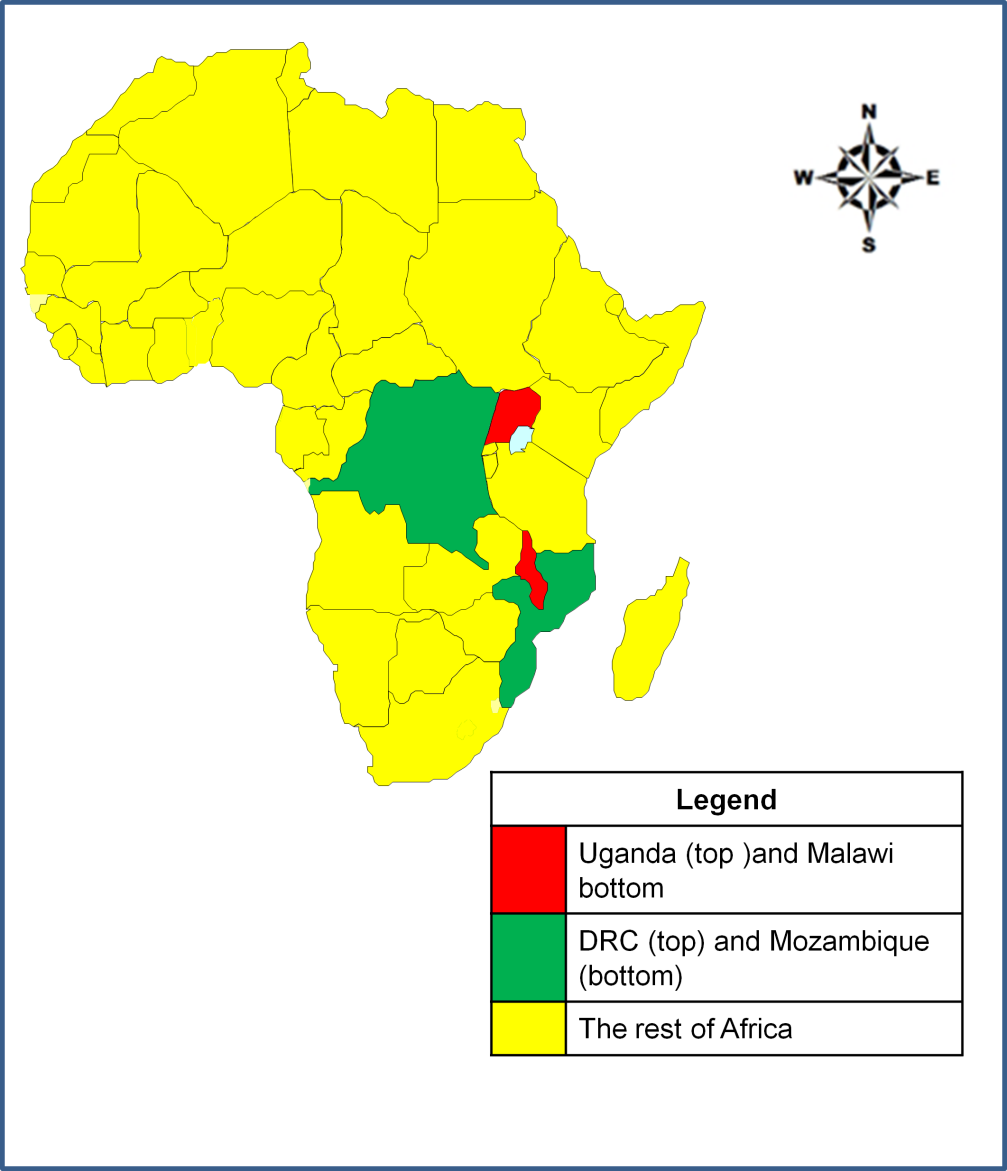
The map of Africa showing the location of Uganda and Malawi and the neighboring countries on the study borders.

This article reviewed data from the Ministries of Health epidemiological records of the two countries in Sub-Saharan Africa namely; Uganda and Malawi which were purposively selected to represent East Africa and Southern Africa regions respectively. The countries were selected because they had endemic cholera outbreaks along their international country borders with Democratic Republic of Congo (DRC) and Republic of Mozambique respectively. The study area is shown in Fig 1.

Democratic Republic of Congo and Republic of Mozambique are among the countries with the highest cholera burden in WHO African region. The two countries (Malawi and Uganda) were both implementing IDSR strategy, thus had similar and comparable data including the use of the same official national language which was English and standard case definition for cholera below [21].

In an area with no cholera epidemic: a suspected case of cholera was defined as a patient aged five (5) years or more presenting with severe dehydration or a death from acute watery diarrhoea.

If there is a cholera epidemic in the area: a suspected case is any person age 5 years or more with acute watery diarrhoea, with or without vomiting. In Uganda, this case definition was modified by lowering the age of the affected person to 2 years or more.

Confirmed cholera case is a suspected case in which *Vibrio cholerae* O1 or O139 has been isolated from the stool sample of the patient.

For the purpose of this study, a cross-border cholera outbreak was defined as a confirmed cholera epidemic occurring in the community located along or close to the international country boundary with cholera patients originating from both countries sharing the international border.

For the two selected countries, records and personal experiences from the most recent cross-border cholera outbreaks were extracted, analyzed and shared. The authors had added advantage because they actively participated in the implementation and coordination of cholera prevention and control interventions in their respective countries.

Additional information to support the data from the line lists were obtained from country specific outbreak reports (weekly reports, outbreak investigation and response reports) for the selected outbreaks meeting inclusion criteria. No interviews were conducted, however, information on the risk factors for the outbreaks, interventions implemented and challenges noted were collected from the outbreak investigation reports and weekly epidemiological reports.

For an outbreak to be included in the study the following had to be met namely; a cholera outbreak occurring in 2015 in the border districts with the patient data showing location (country) of origin from both neighboring countries. We excluded cholera outbreaks reported in the border districts but data, reports and experiences from only one country and nationality. In this article the country refers to a place of origin of the patient.

Data was collected using a questionnaire on following variables: age, sex, country of origin, date of onset of illness, number of stool samples collected from the cases and tested, stool samples that tested positive by culture, risk factors for outbreaks, interventions implemented to control the outbreaks and challenges noted by the cholera actors in controlling the outbreaks. The data was cross-checked for errors before and after entering it into the spreadsheets. Data storage and analysis was conducted using spreadsheet (excel), powerpoint and graphpad by the authors. The maps were drawn using Arc View geographical referencing software. The shapefiles used to draw the maps were downloaded from www.maplibrary.org and from Uganda Bureau of Statistics (UBOS), www.ubos.org/statistical-activities/gis/. The administrative units used to shade the maps were the district level for Malawi-Mozambique and Sub-county level for Uganda-DRC. The difference between the mean ages of the two groups of the reported cross-border cases was tested using unpaired t-test. Ethical consideration was paramount in the conduct of this study. Involvement of the human subjects was limited to the authors describing their personal experiences.

The information used in this study was secondary data, collected during the routine surveillance activities by the ministries of health of the two representative countries (Malawi and Uganda).

Institutional Review Boards (IRBs) of the two representative countries from African sub-regions of Eastern and Southern Africa were consulted and IRBs weavers provided. The authors had access to personal patient information from the cholera line lists (cholera patient registers) namely the patient numbers, names of patients and their villages of origin. However, in this study these personal identifiers were omitted (converted to anonymous) or substituted with the codes known only to the authors or categorization appropriate for the public surveillance information. For instance, the villages were replaced with the sub counties / country as appropriate.

One of the authors (Yosia Baluku) interacted with the patients during his work as a healthcare provider to save lives. This allowed him access to more personal patient information. However, in his contribution to this study he strictly observed the professional ethical requirement (confidentiality, beneficence and informed consent) and only shared information required for routine surveillance work and free from personal identifiers.

The potential benefits of this study to the cross-border cholera affected communities are enormous and include: awareness rising among cholera actors and guidance to the policy-makers to take action to prevent more infections, suffering and deaths.

## Results

### Location of the cross-border outbreaks

**Fig 2:**
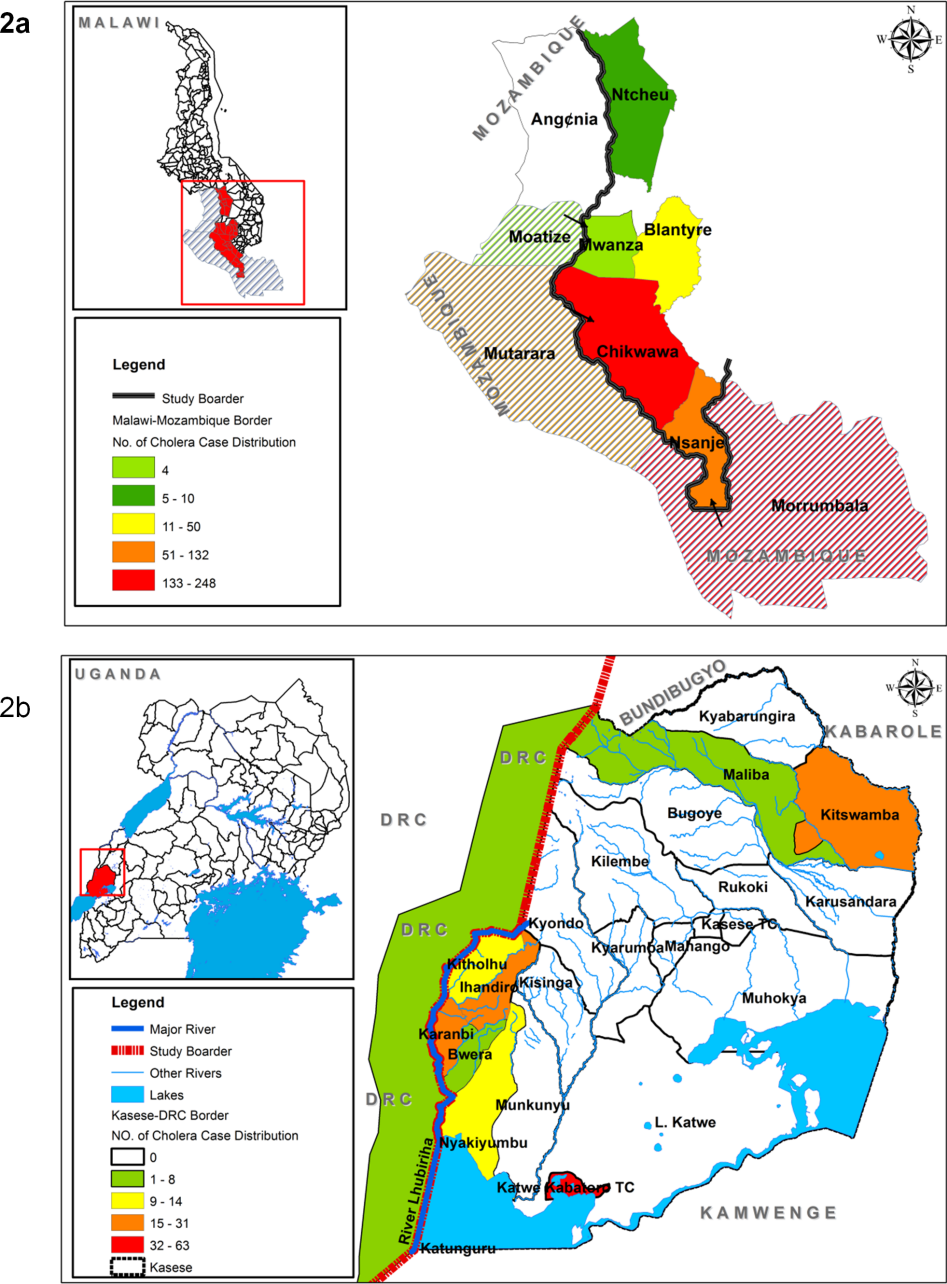
The map of Malawi (2a) and Uganda (2b) showing the location of the cholera affected districts and the distribution of the cholera cases in the study area.

Using the inclusion and exclusion criteria above, out of 5 districts in Malawi and 8 districts in Uganda which reported cholera outbreaks in 2015, 3 and 1 districts respectively were classified as fitting cross-border outbreaks. These districts were: Mwanza, Nsanje and Chikwawa in Malawi and Kasese district in Uganda (Fig 2).

Chikwawa district and Karambi sub-county (Kasese district) were the most affected communities in Malawi and Uganda respectively. In both Malawi and Uganda the outbreaks later spread to other parts of the country leading to 60 cases with 3 deaths, CFR of 5% and 102 cases with 0 deaths respectively. The areas where the outbreaks spread were Blantyre and Ntcheu districts in Malawi and Katwe-Kabatoro Town Council (TC) and Kitchwamba Sub-county in Kasese district, Uganda (Fig 2).

### Weekly surveillance reports

**Fig 3:**
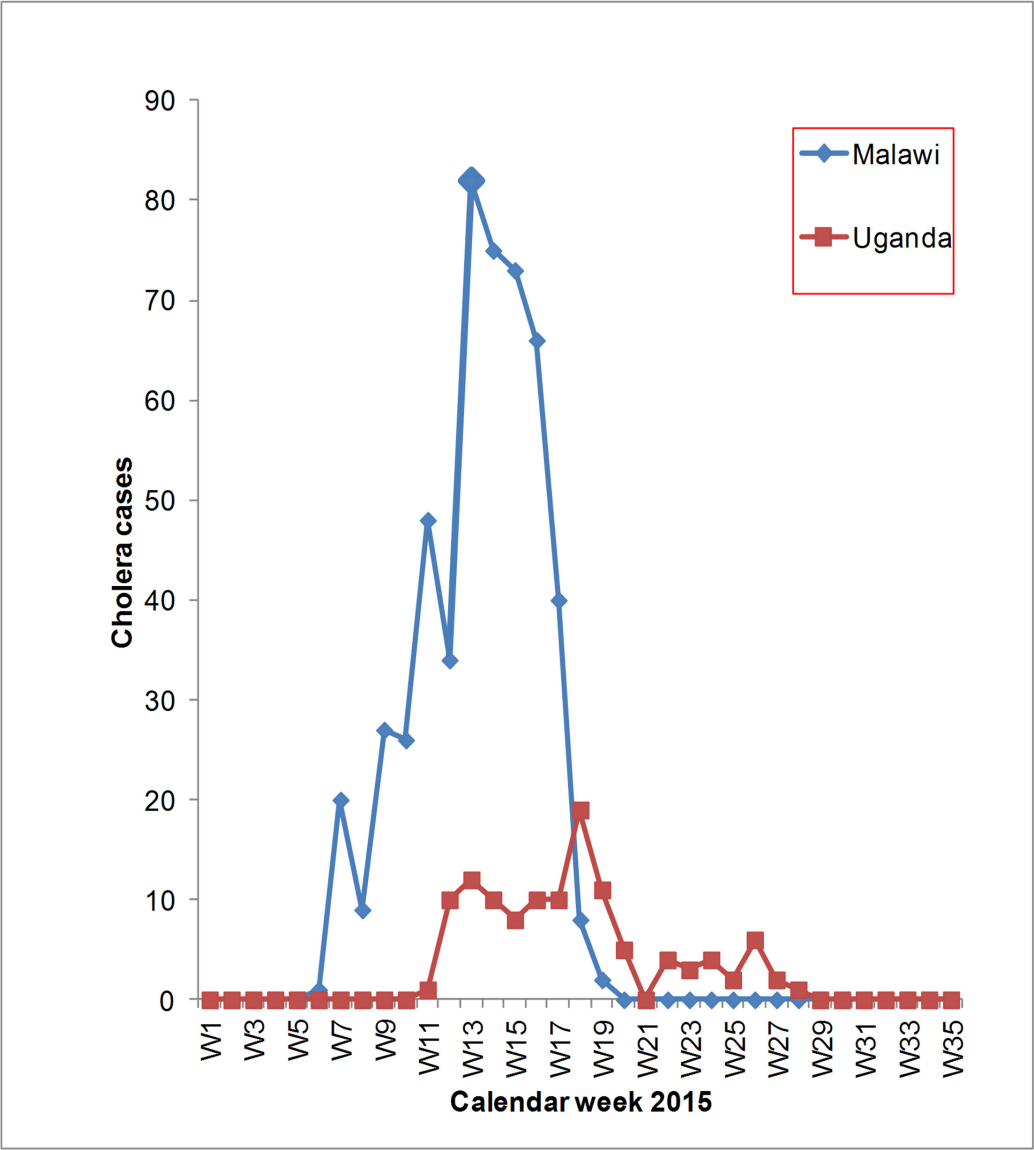
Reported cross-border cholera cases in Malawi and Uganda in 2015.

A total of 603 cholera cases with 5 deaths were recorded in Malawi and Uganda during the cross-border cholera outbreaks involving the communities along the common country borders of Malawi-Mozambique and Uganda-DRC in 2015. Malawi recorded 495 cholera cases with 2 deaths, case fatality rate of 0.6%. While Uganda recorded 118 cholera cases with 2 deaths and case fatality rate of 1.7%. The outbreak in Malawi started in February 2015 (6^th^ calendar week) and ended in May 2015 (20^th^ calendar week). The outbreak in Uganda started in March 2015 (11^th^ calendar week) and ended in July 2015 (28^th^ calendar week). In both countries the outbreaks lasted for over 3 months, 14 weeks for Malawi-Mozambique and 17 weeks for Uganda-DRC border (Fig 3).

**Fig 4:**
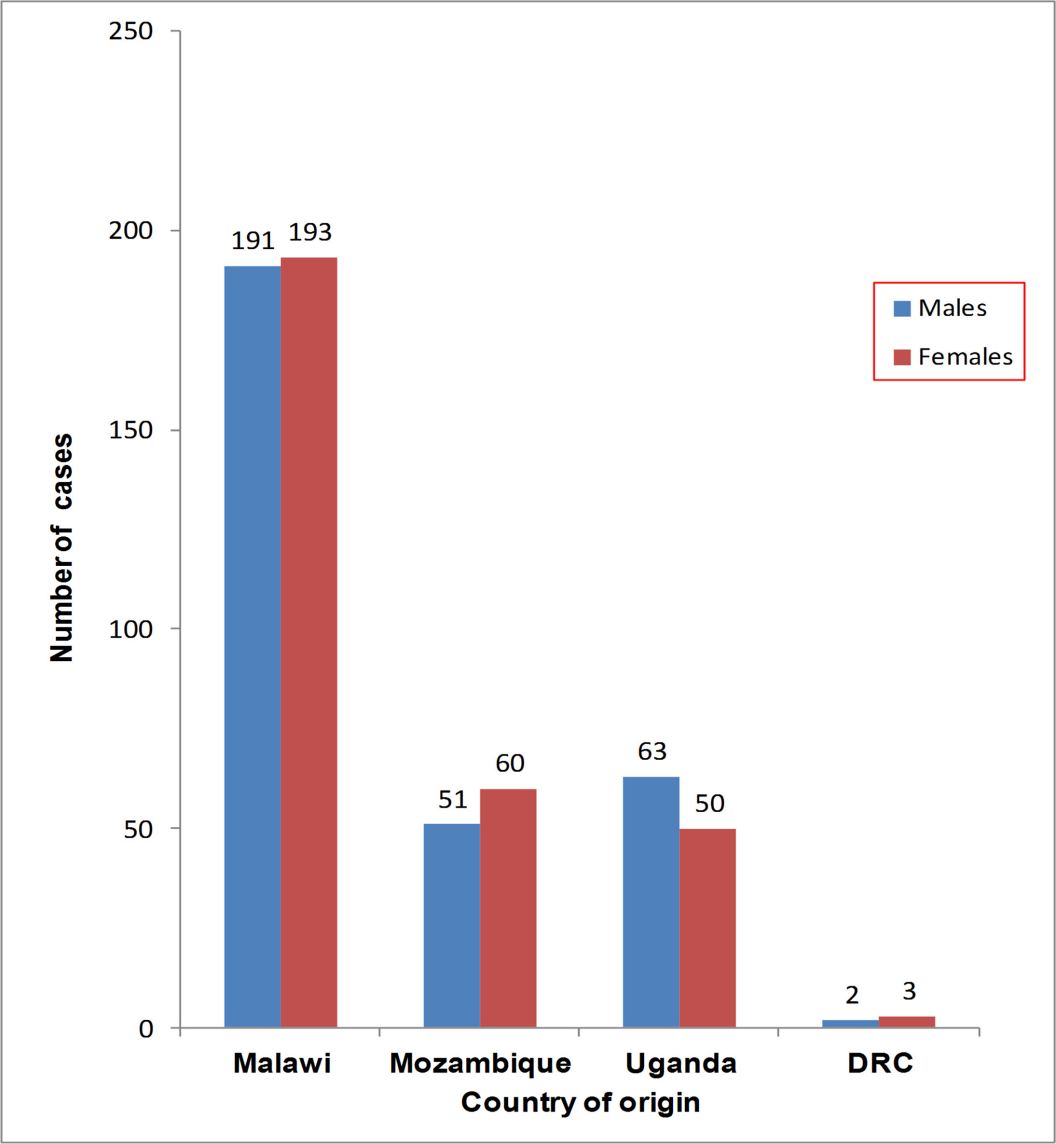
The distribution of cholera cases by country of origin and sex.

These outbreaks peaked in the same month, April 2015 in both regions (Fig 4).

Patients recorded and treated in Malawi and Uganda originated from both sides of the international borders. The host country (Malawi or Uganda) where treatment occurred reported the biggest number of cross-border cholera cases and deaths.

**Fig 5:**
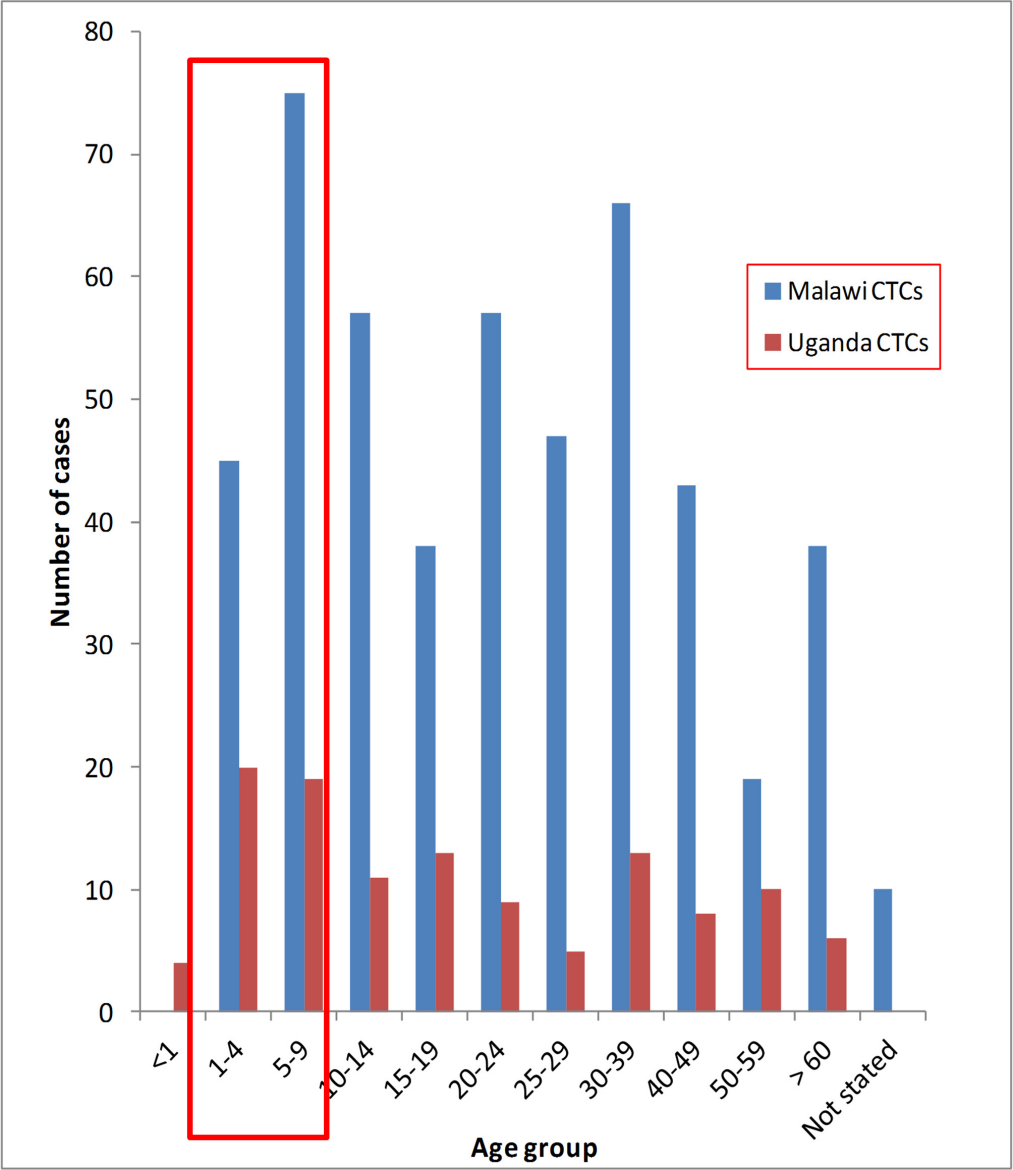
The age distribution of cross-border cholera cases reported in Malawi and Uganda cholera treatment centres in 2015.

Slightly more females were affected in Malawi, Mozambique and DRC than the males. In Uganda, 53.30% (63/118) of the affected were males. The under-fives and school going children combined contributed 24.2% (120/495) and 36.4% (43/118) of all cases seen in the cholera treatment centres in Malawi and Uganda respectively. The distribution of the cross-border cholera cases by age groups in the two regions was as shown in Fig 5.

The mean ages of the cholera cases were 24.54 years, Standard Deviation (SD) of ±18.39 years for Malawi-Mozambique and 22.59 years, SD of ±20.17 years for Uganda-DRC cross-border cholera cases respectively. The mean ages of the two groups of patients (Malawi-Mozambique and Uganda-DRC) were not statistically different (P-value, 0.3341).

### Transmission and sources of cholera infection

The communities along the borders interacted freely with their counterpart on the other side of the international boundary. In Malawi, this was facilitated by the common market regional grouping called Southern Africa Development Community (SADAC) to which the two neighbors belonged. The reasons for migration across the borders were: visiting relatives, cross-border trade, looking for employment and seeking better medical treatment. Informal border crossing was common, with the two international borders acting as an artificial demarcation to the communities.

Besides, the investigation reports indicated that the risk factors for the cholera outbreaks were on the either side of the international borders for both Malawi and Uganda since the residents and migrants acquired cholera on any side of the border.

Also some migrants acquired cholera on the other side of the border and crossed to receive treatment while some could have crossed incubating the infection and developed the disease after crossing into Malawi or Uganda where they ended up getting medical care. Another possibility was that some migrants acquired infection in Malawi or in Uganda after interacting with the sick residents or cholera contaminated environment.

### Laboratory results

A total of 146 stool samples were collected and tested for *Vibrio cholerae* organisms. The two sub-regions had the same *Vibrio cholerae* serogroup O1 biotype El Tor. The serotype on Uganda-DRC border was *Inaba* while that on Malawi-Mozambique border was *Ogawa*. In both settings the pathogens were sensitive to tetracycline antibiotics, a cheaper and more affordable antibiotic. Laboratory results for the tests conducted during the cross-border cholera outbreaks in 2015 in the two sub-regions were as in Table 1.

**Table 1:** Laboratory results of the cross-border cholera cases seen in Uganda and Malawi in 2015.

Country	Total stool samples tested	Number of positive stool samples	Type of organism isolated	Positivity rate	Antibiotic sensitivity pattern
Uganda	74	60	Vibrio cholerae O1 El Tor Inaba	81%	The organisms were sensitive to Tetracycline and Ciprofloxacin antibiotics but resistant to Ampicillin, Chloramphenicol, Cotrimoxazole and Nalidixic acid
Malawi	72	55	Vibrio cholerae O1 El Tor Ogawa	76%	The organisms were sensitive to Doxycycline (Tetracycline), Nalidixic acid, Ciprofloxacin and Erythromycin antibiotics.

### Risk factors for the outbreaks, interventions implemented and challenges experienced

The Ministries of Health, Malawi and Uganda and their respective local teams identified the factors leading to the cholera outbreaks, and implemented activities to prevent and control these outbreaks. In conducting these activities, the country teams experienced some challenges as shown in the Table 2.

**Table 2:** Risk factors for the cross-border cholera outbreaks, interventions implemented and challenges experienced during response in Uganda and Malawi, 2015.

Country	Risk factors for cholera outbreak	Cholera control Interventions implemented	Challenges experienced during implementation of cholera response
**Uganda**	Use of contaminated river water mainly from *River Lhubiriha*. Poor sanitation resulting from wide spread open defecation. Poor food handling, storage and cooking practices especially eating of cold food. Bathing in water sources mainly rivers (*Lhubiriha*). Bad personal hygiene in particular not washing hands with soap. Rampant migration along the country borders with DRC and vise versa. Bad handling of the dead bodies involving opening and touching of the corpse.	Chlorination of water for household use especially for drinking and food preparation. Promotion of eating of hot foods, safe food preparation and boiling of drinking water. Sanitation, safe water chain and hygiene promotion. Inspection and enforcement of hygiene in the community and in the public places (schools, markets, hotels, etc). Health education on cholera prevention and treatment. Disease surveillance with emphasis on early detection and reporting of suspected cases and deaths. Treatment of the sick in the Cholera treatment centres and in Oral rehydration centres. Medical supervision of the suspected cholera burials to limit community contact with the corpse. Restriction of feasting and promotion of infection control through hand washing, disinfection, good sanitation and hygiene practices.	The outbreak was protracted for over 3 months which disrupted other routine services by withdrawing resources (human, logistics and infrastructure). Lack of information sharing between the two neighboring countries (Uganda and DRC). Inadequate risk factor assessment since some patients originated across the border in DRC where the Ugandan health workers could not easily access. Poor communication between the patients and the health workers due to language barrier; the official communication language for the health workers in Uganda was English while that in DRC was French. Lack of collaboration between the two sister governments (cholera actors) in implementation of the cholera response. Rampant movement of the patients and communities across the border which complicated the follow up for risk assessment and exacerbated the spread of the infection. Lack of accurate population data for response planning especially for quantification of supplies for water chlorination, and hygiene. During the 18^th^ and 24^th^ calendar weeks, the outbreak along Uganda-DRC border spread to two different localities in the interior within the same border district leading to 102 cholera cases with no deaths. These outbreaks in the interior were quickly detected and controlled within two weeks of confirmation for each affected location.
**Malawi**	Floods leading to contamination of the water sources. Poor sanitation and hygiene. Use of contaminated water sources. Migration across the Malawi-Mozambique border and vise-versa.	Similar interventions as those implemented in Uganda plus use the of OCV to complement the WASH interventions. A total of 160,000 doses of OCV were imported for vaccination campaign and used for cholera control in Malawi.	Similar challenges as those documented in Uganda were noted in Malawi except the following: the border was Malawi-Mozambique and the official language of communication for the health workers in Mozambique (replacement for DRC) was Portuguese not French. Implementation of OCV required accurate data which was not available since the population in Mozambique which also benefited could not be accurately estimated. The recommended vaccine dose for OCV by WHO is two doses given 14 days apart. However, some clients mainly those from Mozambique received only one dose and could not be located to receive the second dose. The outbreak later spread to other districts in Malawi namely; Blantyre and Ntcheu leading to 60 cases and 3 deaths with high CFR of 5%.

## Discussions

Our study showed that cross-border cholera outbreaks are a major public health threat which affects all categories of people along the common country borders in Eastern and Southern Africa with children being the most affected group. These outbreaks cause protracted ill-health and deaths and can easily spread to other areas with devastating consequences as happened in both Malawi and Uganda.

Despite these, cross-border cholera outbreaks have received little attention and identification by the cholera actors (governments and non-governments). Few studies have documented the experiences of managing cross-border cholera outbreaks in Sub-Saharan Africa. This article is unique in highlighting the importance of this relatively common but marginalized cholera issue. The current cholera prevention and control strategies/guidelines do not identify cross-border cholera outbreaks as a special threat requiring dedicated attention from all actors.

Similarly, though these outbreaks involve both countries this is not conveyed in the national reports submitted to WHO by the countries. Therefore, there is no accurate quantification of the burden of these outbreaks. Also during implementation of the cholera control interventions, the affected countries generally work exclusively within a country rather than in an international manner, and this national approach may lead to protracted outbreaks.

It seems likely that these unilateral efforts within these bordering communities provide a fertile ground and probably a reservoir for cholera multiplication allowing the transmission and spread of cholera to other areas as happened in both sub-regions in this study.

In the two sub-regions the outbreaks were protracted and peaked at the same time, possibly because they were aggravated by similar weather and environmental factors such as increased rainfall which occurred during the month of April 2015 resulting in floods in some areas. This therefore, presents an opportunity for the sub-regions to collaborate and acquire more reliable and accurate early warning equipments for the benefit of the entire region without duplication of the efforts.

According to this study, the most affected age groups in both sub-regions were the children 1–4 years and 5–9 years (school going children). This suggests increased vulnerability of these age groups. In the two study countries of Malawi and Uganda these age groups receive childhood vaccinations (non cholera vaccines) with support from Global Alliance for Vaccine and Immunization (GAVI). These same groups therefore, should be supported to access OCV from the available GAVI stock-piles [22]. To reduce on the operation cost of OCV administration in cholera endemic communities for the 1–4 years and school going children, we propose the integration of OCV into routine vaccination program for the cholera prone border communities.

Furthermore, in order to comprehensively address the gaps noted in this study, concerted efforts by the two neighboring countries is required. These efforts should be in terms of sharing of the health information, joint planning of response activities, coordination and harmonized implementation of cholera control interventions. In addition, the sources of funding for joint cholera prevention and control activities should be clearly specified in the revised strategies so as to adequately guide the country teams. The 2014–2015 Ebola outbreak in West Africa provided good experience for the countries and international bodies regarding the benefits of collaboration in the prevention and control of the outbreak [23]. These good lessons should not be overlooked but should be emulated and used for the prevention, control and ultimately elimination of similar infections with capacity to spread such as cross-border cholera outbreaks.

Though the current cholera outbreak control strategies in Africa such as IDSR / International Health Regulations (2005) [21,24] have improved detection, reporting and response, they are unable to adequately prevent, monitor and control these outbreaks as was shown by this study. Also, the Cholera Compressive Strategy for cholera prevention and control [25] which emphasizes the development of the national plans, integration of OCV in addition to the historical cholera prevention and control approaches such as provision of safe Water, Sanitation and Hygiene (WASH), surveillance, health education, case management among others is very brief or silent in regards to cross-border cholera outbreaks. We think that lack of adequate guidance from these strategies could be one of the main reasons why these outbreaks are common but remain marginalized.

Given this scenario and faced with the threat of cross-border cholera outbreaks, there is urgent need to review the available cholera prevention and control strategies to clearly deliberate on cross-border cholera outbreaks, outline the mechanism for collaboration between states, provide guidance on information sharing between neighbors and propose source of funding for joint activities. The revised strategies should clarify what will be done in case the two affected countries are at war, conflict or are unable to respond on their own.

Most importantly, regional bodies such as the East African Community (EAC), SADAC and international agencies should be empowered to play a more active role in supporting the prevention and control of these outbreaks. In addition, international agencies such as WHO, UNICEF and others should monitor incidences using specific indicators which are able to recognize the trans-boundary (two States) nature of these outbreaks and provided timely support to facilitate the processes (prevention, preparedness and response activities). Where applicable, coordinated implementation of joint interventions leading to prompt prevention and control of these outbreaks should be instituted.

In order for these strategies to be effective, supportive policies have to be enacted at national and sub-regional levels (EAC and SADAC). The signing of bilateral or regional agreements between the countries to ensure implementation of the appropriate measures is paramount. International bodies such as WHO, UNICEF and others could spearhead such efforts to ensure that policies and agreement are in place and implemented.

### Study limitations

We could not estimate the attack rates for each affected community (district or country) during these outbreaks because the other part of the population at risk was not known and belonged to the neighboring country of either DRC or Mozambique. In addition, follow up monitoring after vaccination could not fully be accomplished since some of the persons in Malawi returned to Mozambique immediately after floods had receded.

In this study, characterisation of the pathogens stopped on the phenotying of the *vibrio cholerae* organisms which is inadequate to fully guide interventions. Therefore, further studies especially genotyping should be done to establish similarity and differences in *vibrio cholerae* in the two sub-regions.

Our data analysis relied on the accuracy of the epidemiological records, therefore misclassification and misreporting could not be fully detected. Our epidemic curves may not be the true representation of the outbreaks, since half of the cases and deaths could have been on the other side of the border for which we had no access to information. On the other hand, because we reviewed data and documented experiences from Eastern and Southern Africa only, our findings may not be generalized to entire Sub-Saharan Africa. We therefore recommend that further studies be done in Central and West Africa to better understand the additional barriers to cross-border cholera outbreak prevention and control. Furthermore, a comprehensive study should be conducted to estimate the true burden of these outbreaks in entire Sub-Saharan Africa. The design for this study among other things should be able to compare the disease burden of the cross-border cholera outbreaks with the other cholera outbreaks within individual countries.

## Conclusions

Our study showed that cross-border cholera outbreaks contribute to the high annual reported cholera burden in Sub-Saharan Africa yet they remain silent, marginalized and poorly identified by cholera actors (governments and international agencies). The under-fives and school going children were the most vulnerable groups to these outbreaks.

To successfully prevent and control these outbreaks, guidelines and strategies should be reviewed to provide more focused information, assign clear roles and responsibilities to actors on collaboration, prevention, detection, control, monitoring and financing of joint activities for the cross-border outbreaks.

## Supporting Information

S1 Information Data. (XLS)

S2 Information IRB documents. (ZIP)
