# Supplementary material for: Cross-Border Cholera Outbreaks in Sub-Saharan Africa, the Mystery behind the Silent Illness: What Needs to Be Done?
Source: PLoS One. 2016 Jun 3;11(6):e0156674. doi: 10.1371/journal.pone.0156674 (PMC4892562; doi:10.1371/journal.pone.0156674)
Supplement: S2 Information — (ZIP) [file pone.0156674.s002.zip › S 2 File. IRB documents/S2 File. Cross-border cholera study_ Malawi IRB approval document.pdf]

Telephone: + 265 789 400
Facsimile: + 265 789 431

All Communications should be addressed to:
The Secretary for Health

In reply please quote No. MED/4/36c
MINISTRY OF HEALTH
P.O. BOX 30377
LILONGWE 3
MALAWI

7th March 2016

Maurice M'bangómbe
Ministry of Health
(Epidemiology Unit)

Dear Sir/Madam,

RE: REQUEST TO CONDUCT A REVIEW OF SECONDARY DATA –MOH REPORTS AND SURVEILLANCE ROUTINE DATA ON CHOLERA OUTBREAK IN BORDER DISTRICTS IN 2015

Thank for you for the above titled study that you submitted to the National Health Sciences Research Committee for review.

The committee reviewed the study and **exempted** it from scientific and ethical review **because** it is an outgoing programme existing within the Ministry of Health. **However,** should the researchers wish to publish their findings, they should share with NHSRC.

Kind regards from the Secretariat.

Dr Benson Chilima
CHAIRMAN, NATIONAL HEALTH SCIENCES RESEARCH COMMITTEE
