# Supplementary material for: Cross-Border Cholera Outbreaks in Sub-Saharan Africa, the Mystery behind the Silent Illness: What Needs to Be Done?
Source: PLoS One. 2016 Jun 3;11(6):e0156674. doi: 10.1371/journal.pone.0156674 (PMC4892562; doi:10.1371/journal.pone.0156674)
Supplement: S2 Information — (ZIP) [file pone.0156674.s002.zip › S 2 File. IRB documents/S2 File. Cross-border cholera study_Uganda IRB document.pdf]

MAKERERE UNIVERSITY

P.O. Box 7072 Kampala Uganda
Website: **www.musph.ac.ug**

Tel: *256 414 532207/543872/543437*
Fax: *256 414 531807*

COLLEGE OF HEALTH SCIENCES
SCHOOL OF PUBLIC HEALTH
HIGHER DEGREES, RESEARCH AND ETHICS COMMITTEE

23rd February, 2016

Prof. Christopher Garimoi Orach, PhD
Ugandan Principal Investigator
Cross-border cholera Outbreak Study

Re: **Exemption from IRB review and waiver of requirement of informed consent for a study titled: "Cross-Border Cholera outbreaks in Sub-Saharan Africa, the mystery behind the silent illness: What needs to be done?"**

The Makerere University School of Public Health, Higher Degrees, Research and Ethics Committee received a request for review of the above referenced study for determination of exemption from IRB review and request for waiver of informed consent for the study participants.

It was noted that the study utilized secondary data; including Ministry of Health reports, surveillance data on Cholera outbreaks in border districts.

The study meets the requirement for Exemption from IRB review based on the HDREC SOP section 1.1 which states as below

- section 1.1 D - page 63, states that Research involving the collection of existing data, documents, records, pathological specimens, or diagnostic specimens, if these sources are publicly available or if the information is recorded by the Investigator in such a manner that the research participants cannot be identified, directly or through identifiers linked to the research participants, and it was noted that you coded any personal identifiable information.

Waiver of requirement of Informed consent qualifies based on the HDREC SOPs (#SOP 702) which states that;

- If the research involves no more than minimal risk to the research participants; waiver will not adversely affect the rights and welfare of the research participants; the research could not be practicably carried out without the waiver then the waiver be granted.

The HDREC also requests that you share a **progress report or end of study report/ publication** after completion of this work on wtusiime@musph.ac.ug

Sincerely,

Dr. Suzanne Kiwanuka
Chairperson: Higher Degrees, Research and Ethics Committee
